# Febrile neutropenia in French emergency departments: results of a prospective multicentre survey

**DOI:** 10.1186/cc8972

**Published:** 2010-04-19

**Authors:** Stéphanie André, Pierre Taboulet, Caroline Elie, Noël Milpied, Michel Nahon, Gérald Kierzek, Mariève Billemont, Franck Perruche, Sandrine Charpentier, Hélène Clément, Jean-Louis Pourriat, Yann-Erick Claessens

**Affiliations:** 1 Department of Emergency Medicine, Hôpital Cochin, APHP, 27, rue du Faubourg Saint-Jacques, F-75679 Paris Cedex 14, France; 2 Université Paris Descartes, 12, rue de l'Ecole de Médecine, 75006 Paris, France; 3 Department of Emergency Medicine, Hôpital Saint-Louis, APHP, 1 avenue Claude-Vellefaux, 75010 Paris, France; 4 Department of Biostatistics, Hôpital Necker, APHP, 149 rue de Sèvres, 75015 Paris, France; 5 Department of Haematology, Hôpital Haut-Lévêque, Groupe Hospitalier Sud, CHU de Bordeaux, Avenue de Magellan, 33604 Pessac Cedex, France; 6 Department of Emergency Medicine, Hôpital Necker, APHP, 149 rue de Sèvres, 75015 Paris, France; 7 Department of Emergency Medicine, Hôtel-Dieu, APHP, 1 place du Parvis Notre-Dame, 75004 Paris, France; 8 Department of Emergency Medicine, Hôpital Purpan, CHU de Toulouse, Place du Docteur Baylac, 31059 Toulouse Cedex 9, France

## Abstract

**Introduction:**

Febrile neutropenia (FN) is common in cancer patients receiving myelotoxic therapy. The procedures to treat FN are well established in oncology, but it is unclear whether management is adequate in the emergency department (ED).

**Methods:**

This prospective, multicentre, observational study was carried out in 47 French EDs for 6 months. Patients were adults presenting at the ED with FN after myelotoxic treatment for cancer. Severity of infection was defined according to Bone criteria for severe sepsis and septic shock (SS/SSh) and risk was determined according to Multinational Association of Supportive Care in Cancer (MASCC) criteria. The end point was the implementation of guidelines. Management of patients with SS/SSh required: (i) adequate intravenous (IV) antimicrobial therapy for the first 90 min (broad-spectrum beta-lactam with or without an aminoglycoside); (ii) fluid challenge (500 mL); (iii) lactate measurement; (iv) at least one blood culture; and (v) hospitalization. Management of patients without SS/SSh required: (1) no initiation of granulocyte - cell stimulating factor (G-CSF); (2) adequate IV antimicrobial therapy (broad-spectrum beta-lactam) and hospitalization if the patient was high-risk according to MASCC criteria; (3) adequate oral antimicrobial therapy (quinolone or amoxicillin/clavulanate or cephalosporin) and hospital discharge if the patient was low-risk.

**Results:**

198 patients were enrolled; 89 patients had SS/SSh, of whom 19 received adequate antimicrobial therapy within 90 min and 42 received appropriate fluid challenge. Blood cultures were obtained from 87 and lactate concentration was measured in 29. Overall, only 6 (7%) patients with SS/SSh received adequate management. Among 108 patients without SS/SSh, 38 (35%) were high-risk and 70 (65%) low-risk. In the high-risk group, adequate antimicrobial therapy was given to 31 patients, G-CSF was initiated in 4 and 35 were hospitalized. In the low-risk group, 4 patients received adequate oral antimicrobial therapy, IV antimicrobial therapy was prescribed in 59, G-CSF was initiated in 12 and six patients were discharged. Adequate management was given to 26/38 (68%) high-risk and 1/70 low-risk patients. Factors associated with adequate management were absence of SS/SSh (*P *= 0.0009) and high-risk according to MASCC criteria (*P *< 0.0001).

**Conclusions:**

In this French sample of cancer patients presenting to the ED with FN, management was often inadequate and severity was under-evaluated in the critically ill.

## Introduction

The occurrence of febrile neutropenia in cancer patients should lead to cautious severity assessment in order to provide appropriate management and therefore improve prognosis [1]. Despite specific recommendations, febrile neutropenia is still associated with high morbidity and mortality [2] and elevated health-related costs. The underlying conditions associated with febrile neutropenia make patients more vulnerable. Cancer itself compromises survival [3], impairs innate and adaptative immunity, and patients have a higher chance of developing a nosocomial infection [4]. Therefore, severe infections are common in this population. To help physicians safely decide the site of care for patients with febrile neutropenia, criteria have been determined [5], and sensitive scoring systems have been validated to limit patients' misclassification [6]. In this setting, Multinational Association for Supportive Care in Cancer (MASCC) criteria were developed to help physicians make decisions about the site of care and overall management of patients with febrile neutropenia. This score mainly relies on subjective criteria such as the evaluation of clinical symptoms and hydration state.

Sepsis, severe sepsis and septic shock were defined 15 years ago as a continuum of increasing severity of the host response to the pathogen [3] closely related to predisposition, organ failure and systemic response, and to the microorganism and site of infection [7]. Management of septic patients has significantly improved over the past decade as a result of consensus guidelines published by 11 scientific societies [8-10]. These recommendations have been widely disseminated, but detecting patients at risk remains a daily challenge in emergency medicine.

The presence of cancer may impair the prognosis of acute patients [8], including septic patients [11] visiting the emergency department (ED) [12]. Reports on the management of febrile neutropenia in EDs are scarce, retrospective and mainly single centre studies. Interestingly, a French survey based on declarative questionnaires reported that 1 out of 31 cancer teams involved emergency physicians in their organizational strategy to manage patients with febrile neutropenia [13]. As the incidence of sepsis has increased in the general population [14], more patients visit the ED for this reason [15] and this had led to a dramatic increase in number of cancer patients in this setting [16].

This study was carried out to describe the management of patients with febrile neutropenia in EDs and to determine how management complies with recommendations. The secondary objective was to determine the factors associated with adequate management.

## Materials and methods

### Study design and ethics

This was a prospective multicentre study carried out in 47 French EDs over a six-month period (4 February to 4 August, 2008). The inform consent from patients was required for this study. The study protocol and patient information procedures were approved by the institutional review board for the protection of human subjects of the Cochin Port-Royal (Paris, France).

### Recruitment of patients

Patients were included if they were adults (>18 years old) who presented at a participating ED with febrile neutropenia after myelotoxic treatment for cancer. Delay between last cytotoxic treatment and occurrence of febrile neutropenia was not pre-specified to enter the study. Definition of febrile neutropenia consisted of a white blood cell count less than 1,000/μL (or neutrophils <500/μL), with a core temperature above 8.3°C (or >38°C on two consecutive occasions). Patients who presented with febrile neutropenia in another setting or who refused to participate were not included in the study.

### Procedure and data collection

An investigator (SA) contacted by phone the team leader of each participating centre to describe the study and explain criteria for eligibility. Each team leader gave the information about this study to the ED team. The physician on duty invited eligible patients to participate and implemented the electronic form. As this was an observation study, data were collected on the basis of usual practices.

The characteristics of each participating centre were recorded, with special reference to the management of febrile neutropenia. Data collected for each patient included demographic characteristics, physical data and medical history focusing on cancer and outcome (discharge, admission, admission to an ICU, death).

### Study objectives

The primary objective was to describe the management of patients with febrile neutropenia in EDs and to determine whether management complies with recommendations. The secondary objective was to determine the factors associated with adequate management.

Evaluation criteria in the guidelines were identified to accurately assess the primary end points. Patients with febrile neutropenia were divided into two groups: those with and those without severe sepsis or septic shock (SS/SSh).

Patients with SS/SSh were selected according to the following criteria [9,17]: blood lactate more than 4 mmol/L, or low blood pressure before fluid challenge (systolic blood pressure <90 mmHg or 40 mmHg below usual systolic blood pressure), or at least one organ dysfunction (pulse oxymetry [SpO_2_] <95% with fraction of inspired oxygen >0.5, blood creatinine >176 μmol/L or oliguria, international normalised ratio >2, bilirubinemia >78 μmol/L, Glasgow Coma Scale <15). Thrombopenia was excluded from the criteria because of the potential effect of chemotherapy on platelet counts. Patients without SS/SSh were identified as high risk or low risk according to the MASCC classification [6] (Table 1). Of note, data that allowed determination of MASCC and presence of SS/SSh were collected at the bedside by attending physicians. Implementation of the database was not intended to help physicians detecting the severity of a patient's condition.

**Table 1:** Classification according to the Multinational Association for Supportive Care in Cancer(MASCC) [6]

Variables	Points (Low-risk if score >20)
Burden of illness	
Age < 60 years	2
Outpatient status	3
No chronic obstructive pulmonary disease	4
No previous fungal infection	4
Clinical state at admission	
No or mild symptoms	5
Moderate symptoms	3
Systolic blood pressure <90 mmHg	5
No dehydration needing perfusion	3

Implementation of the following guidelines was assessed. Management of patients with SS/SSh required for the 90 first minutes [9,10]: (i) a dose regimen of adequate (broad-spectrum) intravenous antimicrobial therapy; (ii) fluid challenge (500 mL) if mean arterial blood pressure was less than 65 mmHg; (iii) lactate measurement; (iv) at least one blood culture; and (v) hospitalization. Management of patients without SS/SSh who were high risk according to MASCC criteria required [18-20]: (i) adequate intravenous antimicrobial agent (broad-spectrum beta-lactam with or without an aminoglycoside); (ii) no initiation of granulocyte-cell stimulating factor (G-CSF); and (iii) hospitalization. Management of patients without SS/SSh who were low-risk according to MASCC criteria required [18-20]: (i) adequate oral antimicrobial agent (quinolone or amoxicillin/clavulanate or cephalosporin); (ii) no initiation of G-CSF; and (iii) hospital discharge.

Patients were divided into two groups; those managed according to recommendations and those who were not, irrespective of initial severity. The two populations were compared to determine the factors associated with adequate management.

### Statistical analysis

Quantitative variables are described as the mean ± standard deviation or median (range) and quantitative variables as number and percent. The adequacy of management according to recommendations was determined for the two sub-groups using Pearson's chi-squared test or Fisher's exact test for qualitative variables and the Student's t test or Wilcoxon rank sum test for quantitative variables. All tests were two-sided. A *P *value of less than 5% were considered statistically significant. All statistical analyses were carried out using R software (Vienna University of Economics and Business, 1090 Vienna, Austria).

## Results

### Emergency departments

The 47 participating EDs were distributed across France and were representative of each metropolitan region. Thirteen (28%) centres were tertiary teaching hospitals, six (13%) were in the Paris area and 29 (60%) had a dedicated unit for cancer patients. The median number of hospital beds was 500 (range, 150 to 2900) and the median number of ED visits was 17,679 during the six months of the study (range, 3,000-39,045). A written procedure for the management of febrile neutropenia was present in 19 EDs (40%) and was formalised with oncologists/haematologists in 15 (32%). This procedure referred to protective isolation in 10 (21%), antimicrobial agents in 16 (34%) and growth factors in 5 (11%) EDs.

### Study participants and febrile neutropenia

**Figure 1:**
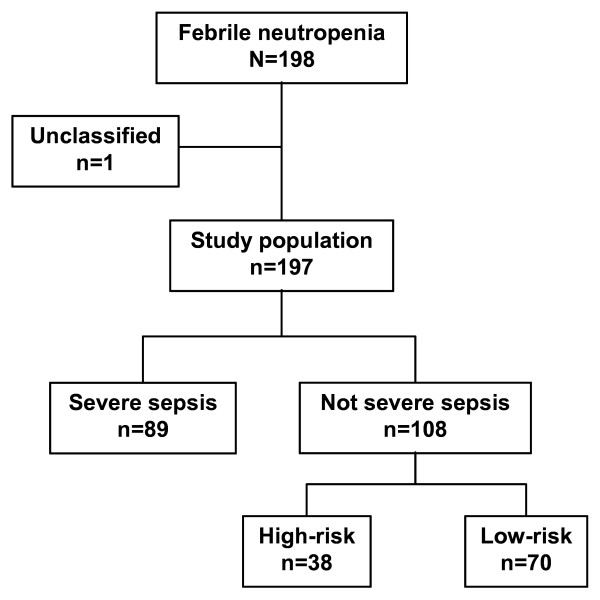
**Flow chart of patients included in the study**.

Among the 777,876 patients who visited the EDs during the study period, 198 fulfilled the inclusion criteria (mean age 61 ± 14 years, 116 (60%) male) corresponding to one case every 3,930 visits; all these patients accepted to participate (Figure 1). Thirteen centres included five patients or more (Tables 2 and 3).

**Table 2:** Characteristics of participating centres

Variables	Number (%) Mean (range)
Participating centres:	47
Tertiary teaching hospitals	13 (27%)
General community hospitals	35 (74%)
Number of beds in hospital	500 (150-2,900)
Number of ED visits during the study period	17,679 (3,000-39,045)
Number of included patients:	198
Tertiary teaching hospitals	111 (56%)
General community hospitals	87 (44%)
Number of centres with ≥ 5 patients	13 (27%)
Written procedure for the management of febrile neutropenia	19 (40%)
Formalised with oncologists/haematologists	
Protective isolation	15 (79%)
Antimicrobial agents	10 (53%)
Prescription of G-CSF	16 (84%)

ED, emergency department; G-CSF, granulocyte-cell stimulating factor.

**Table 3:** Details of location, activity, inclusions and dedicated unit for cancer patients in participating centres

Hospital centres	Number of inclusions	Number of ED visits during study period	Presence of cancer unit	Management procedure for febrile neutropenia
(01) Ain: CH Bourg-en-Bresse	2	15,500	Yes	Yes
(03) Allier: CH Vichy	1	15,700	No	No
(06) Alpes Maritimes: CHU Nice	2	34,300	Yes	No
(07) Ardèche: CH Annonay	2	11,000	No	Yes
(07) Ardèche: CH Aubenas	1	ND	Yes	No
(09) Ariège: CH Val d'Ariège	1	13,200	No	No
(13) Bouches-du-Rhône: CH Martigues	3	16,991	No	No
(15) Cantal: CH Aurillac	2	12,444	Yes	No
(15) Cantal: CH Mauriac	1	3,000	No	No
(17) Charente-Maritime: CH La Rochelle	3	21,500	No	No
(17) Charente-Maritime: CH Rochefort sur mer	3	10,500	ND	ND
(19) Corrèze: CH Tulles	1	9,000	No	No
(22) Côtes d'Armor: CH St Brieuc	2	22,500	Yes	Yes
(24) Dordogne: CH Périgueux	2	14,900	No	Yes
(26) Drôme: CH Valence	5	19,130	Yes	Yes
(28) Eure-et-Loire: CH Chartres	2	19,336	Yes	No
(35) Ile-et-Vilaine: CH St Malo	1	18,000	Yes	Yes
(36) Indre: CH Le Blanc	1	4,570	ND	ND
(37) Indre-et-Loire: CH Chinon	3	6,857	Yes	Yes
(37) Indre-et-Loire: CHU Tours	8	24,000	Yes	Yes
(38) Isère: CHU Grenoble	5	35,600	Yes	Yes
(40) Landes: CH Mont-de-Marsan	5	ND	Yes	No
(42) Loire: CHU St Etienne	13	20,232	Yes	No
(44) Loire Atlantique: CH Chateaubriant	1	7,000	No	No
(45) Loiret: CH Montargis	4	15,222	Yes	Yes
(45) Loiret: CH Orléans	6	19,500	Yes	No
(47) Lot-et-Garonne: CH Agen	1	10,000	No	No
(49) Maine-et-Loire: CHU Angers	4	23,882	Yes	Yes
(54) Meurthe-et-Moselle: CH Lunéville	1	8,200	No	No
(59) Nord: CH Dunkerque	6	26,156	Yes	Yes
(62) Pas de Calais: CH St Omer	1	ND	ND	ND
(63) Puy de Dôme: CH Thiers	1	6,000	No	No
(63) Puy de Dôme: CHU Clermont Ferrand	4	22,000	Yes	No
(64) Pyrénnées Atlantiques: CH Bayonne	2	15,000	Yes	Yes
(64) Pyrénnées Atlantiques: CH Pau	2	13,000	Yes	No
(71) Saône-et-Loire: CH Mâcon	1	17,025	Yes	ND
(72) Sarthre: CH Le Mans	1	28,643	Yes	Yes
(75) Paris: CHU Cochin	22	23,368	Yes	Yes
(75) Paris: CHU Hôtel Dieu	3	22,586	Yes	Yes
(75) Paris: CHU Pitié	9	39,045	Yes	No
(75) Paris: CHU Saint Antoine	1	23,710	Yes	Yes
(75) Paris: CHU Saint Louis	38	16,948	Yes	Yes
(75) Paris: CHU Tenon	5	22,261	No	No
(79) Deux Sèvres: CH Niort	1	17,700	Yes	No
(81) Tarn: CH Albi	3	14,587	No	No
(91) Essonne: CH Longjumeau	6	15,000	Yes	Yes
(94) Créteil: CHU Henri Mondor	5	22,783	Yes	No

ED, emergency department, ND, not determined.

A solid neoplasm was reported in 111 patients (56%) and haematological cancer in 87 (44%). Seventy-four patients (39%) had an underlying disorder. Patients often self-referred to the ED (n = 87, 44%). Forty-seven (24%) patients were treated with G-CSF to prevent neutropenia and 174 (88%) had one or more risk factors that should have prompted the prophylactic use of G-CSF (Table 4).

**Table 4:** Prescription of G-CSF

Variables	Number (%)
Prophylactic prescription of G-CSF before referral in ED	47 (24)
Age >65 years	14 (30)
Recurrent or resistant cancer	28 (60)
Chemotherapy at high-risk for neutropenia (risk >20%)	15 (38)
Previous history of febrile neutropenia	15 (32)
Prescription of G-CSF initiated in ED	27 (19)
Patients with severe sepsis	12 (44)

ED, emergency department; G-CSF, granulocyte-cell stimulating factor.

The characteristics of the patients are summarised in Table 5. Median delay between chemotherapy and ED visit was 10 days, ranging from 4 to 35 days. According to the criteria selected for disease severity, 89 (45%) patients had SS/SSh, 108 (55%) did not have SS/SSh and one could not be classified.

**Table 5:** Characteristics of the patients

	Total population	Patients with severe sepsis	Patients without severe sepsis	*P*
Number of patients	198	89	108	
Age (years), mean ± SD	61 ± 14	65 ± 13	57 ± 14	<0.001
Female, n (%)	79 (41)	30 (34)	49 (46)	0.11
Karnofsky index, median (range)	70 (30-100)	70 (30-100)	80 (30-100)	0.06
Underlying disorders, n (%)	73 (38)	36 (43)	37 (35)	0.32
Chronic pulmonary disease	9 (12)	6 (16)	3 (3)	
Chronic heart failure	12 (16)	9 (24)	3 (3)	
Cirrhosis	7 (9)	4 (11)	3 (3)	
Hemodialysis chronic renal failure	2 (3)	2 (5)	0	
Severe neurological disorder	3 (4)	1 (3)	2 (2)	
Other	51 (69)	21 (57)	30 (29)	
Haematological neoplasm, n (%)	87 (44)	40 (45)	47 (44)	0.84
Lymphoproliferation	64 (32)	30 (34)	34 (31)	
Myeloproliferation	22 (11)	10 (11)	12 (11)	
Undetermined	1	0	1	
Solid cancer	111 (56)	49 (55)	61 (56)	
Lung	39 (20)	24 (27)	15 (14)	
Breast	26 (13)	9 (10)	17 (16)	
Urological and genital	18 (9)	9 (10)	9 (8)	
Gastro-intestinal	13 (7)	5 (6)	7 (6)	
Other or undetermined	15 (8)	1	8 (7)	
Presence of metastasis or uncontrolled	133 (67)	69 (78)	63 (58)	0.004
Previous history of febrile neutropenia	60 (31)	24 (27)	36 (35)	0.32
Chemotherapy at high-risk for neutropenia	36 (23)	15 (21)	21 (25)	0.57
Corticosteroids	83 (42)	37 (42)	46 (43)	0.89
Prophylaxis with G-CSF	47 (25)	18 (20)	29 (28)	0.22
Antimicrobial therapy prior to ED	48 (25)	15 (17)	33 (31)	0.03
MASCC <20 (high-risk)	105 (53)	67 (75)	38 (35)	<0.001

Results are expressed as number (%), mean ± standard deviation (SD), or median (range). *P *values below 0.05 are statistically significant.

ED, emergency department; G-CSF, granulocyte-cell stimulating factor; MASCC, Multinational Association for Supportive Care in Cancer.

Among the 89 patients with SS/SSh, ED physicians recognised the severity signs in 45 (55%). Blood cultures were obtained from 87 (98%) patients and lactate concentration was measured in 29 (32%). Antimicrobial therapy with a broad-spectrum beta-lactam was started within 90 minutes in 19 of 86 (22%) patients (data missing for three). Among these patients, nine (10%) also received an aminoglycoside. Appropriate fluid challenge was given to 43 (49%) patients and 88 were hospitalised (99%), including 18 who were admitted to the ICU. Only six (7%) patients with SS/SSh received adequate management (Table 6). Of note, G-CSF was initiated in the ED in 12 patients (14%) with SS/SSh.

**Table 6:** Characteristics of the management of febrile neutropenia in patients with or without severe sepsis

Management in the ED	Patients with severe sepsis	Patients without severe sepsis	
	(n = 89)	High risk (n = 38)	Low risk (n = 70)
Adequate antimicrobial therapy	28 (32)	30 (81)	31 (44)
Supportive treatment			
Fluid challenge	43 (49)	5 (14)	6 (9)
Vasoactive drugs	6 (7)	0 (0)	0 (0)
Laboratory data			
Lactate concentration	29 (33)	1 (3)	11 (16)
Blood cultures	87 (99)	36 (100)	63 (93)
New prescription of G-CSF	12 (14)	4 (11)	12 (17)
Adequate orientation	88 (99)	35 (95)	6 (9)
Global adequate management	6 (7)	26 (68)	1 (1)

Risk of patients without severe sepsis was determined using MASCC criteria (low risk if MASCC ≥ 20; high risk if MASCC <20). All results are expressed as number (%). ED, emergency department; G-CSF, granulocyte-cell stimulating factor; MASCC, Multinational Association for Supportive Care in Cancer.

There were 108 patients without SS/SSh: 38 were high risk (35%) and 70 were low risk (65%) according to MASCC criteria. In the high-risk category, adequate antimicrobial therapy was given to 31 (81%) patients, G-CSF was initiated in the ED in four (10%) and 35 (95%) were hospitalised. In the low-risk category, four (6%) patients received an adequate oral antimicrobial agent, but an IV antimicrobial agent was prescribed in 59 (84%) cases. G-CSF was initiated in the ED in 12 (17%) patients. Only six patients (9%) were discharged; the remaining patients were believed to have been admitted. Adequate management was proposed in 27 of 108 (25%) patients without SS/SSh, 26 (68%) high-risk patients and one low-risk patient (Table 2).

The 33 (17%) patients who were given adequate management were compared with the 161 (83%) patients managed inadequately (important data were missing in four patients that were excluded from the analysis). Patients without SS/SSh were significantly more likely to receive adequate management than those with SS/SSh (*P *= 0.00009). On the other hand, adequate management was proposed in 32 of 103 high-risk patients (31%) whereas only 1 low-risk patient out of 91 (*P *< 0.0001) was treated according to recommendations (Table 7).

**Table 7:** Factors associated with adequate management

Variables		Adequate management (n = 33)	Inadequate management (n = 161)	*P*
Tertiary teaching hospital	Yes	24 (12)	92 (88)	0.1
	No	9 (21)	68 (79)	
Number of ED visits during study period	<20 000	17 (18)	78 (82)	0.80
	≥ 20 000	15 (16)	76 (84)	
Unit for cancer patients	Yes	29 (18)	132 (82)	1
	No	4 (15)	22 (85)	
Written procedures for febrile neutropenia management	Yes	24 (21)	92 (79)	0.16
	No	9 (13)	62 (87)	
Age (mean ± SD)		58 ± 17	61 ± 14	0.30
Sex	Male	17 (15)	97 (85)	0.29
	Female	16 (21)	61 (79)	
Karnosfsky index (median (range))		70 (30-100)	80 (30-100)	0.07
Place of stay	Home	30 (18)	139 (82)	0.23
	House care	2 (40)	3 (60)	
Underlying disorder	Yes	12 (17)	60 (83)	0.78
	No	21 (18)	94 (82)	
Type of cancer	Haematological	14 (17)	70 (83)	0.91
	Solid cancer	19 (17)	91 (83)	
Presence of metastasis or uncontrolled	Yes	22 (17)	108 (83)	0.96
	No	11 (17)	53 (83)	
Antimicrobial therapy prior to ED	Yes	9 (9)	39 (91)	0.75
	No	24 (17)	119 (83)	
Time of ED visit	Day	18 (15)	102 (85)	0.24
	Night	15 (22)	54 (78)	
Level of nurse triage	<3	20 (22)	73 (78)	0.43
	≥ 3	8 (16)	42 (84)	
Presence of severity signs according to ED physician	Yes	9 (17)	45 (83)	0.82
	No	24 (18)	109 (82)	
Severe sepsis	Yes	6 (7)	80 (93)	0.0009
	No	27 (25)	81 (75)	
Cancer specialist advice	Yes	14 (17)	70 (83)	0.95
	No	18 (17)	88 (83)	
MASCC classification	High risk	32 (31)	71 (69)	<0.001
	Low risk	1 (1)	90 (99)	
Protective isolation	Yes	29 (19)	128 (81)	0.50
	No	4 (13)	26 (87)	
Surgical management	Yes	2 (50)	2 (50)	0.12
	No	26 (15)	146 (85)	
Adequate orientation	Agree	26 (17)	129 (83)	0.65
	Disagree	7 (20)	28 (80)	

Values shown are number and percentage unless stated otherwise. *P *values below 0.05 are statistically significant.

ED, emergency department; G-CSF, granulocyte-cell stimulating factor; MASCC, Multinational Association for Supportive Care in Cancer; SD, standard deviation.

## Discussion

The results of this study suggest that cancer patients with febrile neutropenia visiting the ED are likely to present with SS/SSh, and their management does not comply with current guidelines especially in those with more severe infections.

The 47 participating centres were distributed across France and presented heterogeneous characteristics. This was representative of French EDs overall. As only 60% of hospitals had a dedicated cancer unit, it was assumed that cancer patients with febrile neutropenia visited community hospitals with this acute adverse event. This suggests that procedures to treat these patients should be available in every ED. However, only 40% of participating centres reported a written protocol for the management of febrile neutropenia. A total of 198 consecutive patients with febrile neutropenia were included in the study. The demographic characteristics of these patients corresponded to epidemiologic data published by the French Institute Survey for cancer [16].

EDs are first dedicated to the management of patients with severe disorders including severe infections. A recent study reported that SS/SSh accounted for more than 500,000 visits annually in US EDs [21]. In addition, the incidence of severe sepsis is increasing [22], cancer is a predisposing factor for sepsis, and the number of cancer patients will almost double within one decade [23]. Optimising prevention of febrile neutropenia is therefore an important part of the management. Forty-seven (24%) patients were treated with G-CSF to prevent febrile neutropenia, whereas 174 (88%) had one or more risk factors that should have prompted the prophylactic use of G-CSF [24]. In our sample, there was an under-use of G-CSF in patients at risk of febrile neutropenia. The under-use of G-CSF in oncology practice was also reported previously by Hayes [25]. We therefore believe that emergency physicians will have increasing chances to treat febrile neutropenia.

In their series used to derive and validate the Mortality in Emergency Department Sepsis (MEDS) score, Shapiro and colleagues reported that 35.5% and 2.5% of patients visiting the ED with infection had severe sepsis and septic shock, respectively [12]. Here we reported that 89 (45%) patients with febrile neutropenia presented with SS/SSh. This underscores that chemotherapy-related neutropenia in cancer patients is a risk factor for developing severe infection. In our series, very few patients that developed severe infections were treated according to current guidelines. Indeed, adequate management was initiated in only six patients. This may suggest that detecting severe infections is challenging for emergency physicians. Initial severity assessment is sometimes falsely reassuring and patients may worsen during their stay in the ED [26]. A study conducted in Brazil [14] reported that ED physicians were able to detect severe infection in 15.8% of cases. Implementation of the Surviving Sepsis Campaign guidelines improved detection of severe infections but 61.5% of patients remained under-treated because of inadequate assessment. Measuring lactate concentration has been recommended to help physicians detect [26] and manage severe infections [27]. We observed that lactate was infrequently measured in the present series. Therefore, procedures to optimise detection of severe infection were partially applied in our patients that did not seem to be perceived as severely ill.

A burden of evidence supports the paramount role of early recognition and prompt management of severe infection, and admission to the ICU when applicable [27]. The prognosis of patients with severe infection actually depends on their initial management; that is, treatment received in the ED for half of patients [28]. We observed that few patients received adequate antimicrobial therapy or fluid challenge in an appropriate time-span. We therefore conclude that patients with severe infection were under-treated. Similar findings were reported in a large Spanish study [29], as an incredibly low rate of patients admitted with SS/SSh received process-of-care according to bundles, even after an educational program involving physicians and nurses of the ED and ICU. An inadequate initial assessment may result from difficulties to correctly implement guidelines in a busy ED. First, equipment and physicians' skills to provide complex technical procedures to patients vary betwen hospitals [30]. Barriers can also be related to time consumption of procedures necessary to implement procedures for severe infections. In addition, all team leaders are not fully confident in guidelines to treat severe infections [31]. However, we checked fluid loading and delay to first antimicrobial agent that do not require specific skills or organisation. We observed that these basic treatments were not correctly delivered. In a series of sepsis with hypotension, the delay to antimicrobial agents was over six hours in more than half of patients because infection was not recognised. We believe that most patients were not treated according to guidelines because initial assessment failed to detect the severity of disease.

Despite the efforts in the past decade to produce and distribute specific guidelines for treating severe infection, difficulties persist to detect SS/SSh even in typically at-risk patients such as those with febrile neutropenia.

Delay to first antimicrobial agent has an impact on prognosis in patients presenting infection with severity criteria [32]. Guidelines to treat patients with SS/SSh endorse that first dose of antibiotics should be given in a timespan shorter than 90 minutes [9]. Whereas it can be assumed that earlier antimicrobial agents would improve prognosis in febrile neutropenia, no evidence can currently lead to any recommendations about delay. Consequently, guidelines to treating patients with febrile neutropenia are not clear regarding delays to treatment; therefore, objectives are easier to obtain. This may partly explain why management of patients without SS/SSh frequently reached goals.

A puzzling result is that supportive care was not modified by the intervention of the oncologist or haematologist: the presence of a medical unit dedicated to cancer in the same hospital, the existence of written procedures about febrile neutropenia, or the oncologist's advice did not improve the quality of care. Despite recent validation studies, the relevance of MASCC to guide site of care can be limited because several cornerstone items are missing from this evaluation tool [18]. This supports the fact that the assessment of severity of infection in a short time-span appears to be particularly challenging in onco-haematological patients [33].

The study has several limitations. Whereas simplicity of the study design presumably improved acceptability and feasibility, we cannot rule out that patients could be missed because making clinical research around the clock is sometimes difficult in busy EDs. Our study did not follow up the patients. It was decided to carry out a descriptive study and patients' outcomes were not recorded. Thus, it is unknown whether the prognosis of the patients with febrile neutropenia would have changed if recommendations had been implemented. In addition, the study was proposed to 350 French hospitals, but only 47 took part. Therefore, results could have been biased because EDs that participated were possibly more involved in the management of patients with sepsis and/or cancer. However, these centres were geographically distributed across France and were representative of French EDs because they were mostly set in general hospitals. In addition, only 19 Eds had a written procedure for febrile neutropenia. The convenience series was also limited because no patient waived the invitation to participate in this prospective study. Another limitation was the use of MASCC criteria to decide on the site of care and antimicrobial therapy because this score has never been validated in the ED setting. Stratification of patients using the MASCC scoring system is debatable as consensus meetings suggest that it is not superior to expert advice. However, the MASCC classification has been regularly used as a gold standard to stratify patients with febrile neutropenia. In addition MASCC calculation depends on the burden of the onco-haematological disorder. However, it is usually accepted that patients with advanced cancer under-estimate the severity of the disorder [34,35]. As emergency physicians usually obtain past medical history from patients' interviews, they may misclassify severity of febrile neutropenia when assessed by MASCC level. Finally, we found that patients with more severe and mild febrile neutropenia were inadequately treated according to a univariate analysis. We were unable to identify risk factors for inadequate management by a multivariate analysis, partly because of the size of the sample.

## Conclusions

Patients with febrile neutropenia who visit the EDs are likely to develop severe infection. In this sample, patients who met our definition of SS/SSh had a low rate of being treated with adequate fluid and a low rate of evaluation with serum lactate level. Patients who were considered to be low risk were often admitted to the hospital rather than being discharged home on oral antibiotics. More work is needed within the standard operation protocols of EDs as well as outcome-based research to optimise care for these patients.

## Key messages

• Patients with febrile neutropenia are likely to present to the ED with severe infections.

• More severe patients are poorly recognised and under-treated.

• Patients with mild disorders are over-treated.

• Patients with febrile neutropenia presenting to the ED are usually not treated according to guidelines.

## Abbreviations

ED: emergency department; G-CSF: granulocyte-cell stimulating factor; MASCC: Multinational Association of Supportive Care in Cancer; SS: severe sepsis; SSh: septic shock.

## Competing interests

Yann-Erick Claessens received fees from Amgen France. All other authors declare that they have no competing interests.

## Authors' contributions

SA contributed to the conception, design, and drafting of the manuscript. PT contributed to collection of the data. CE contributed to interpretation and analysis of data and drafting the manuscript. NM contributed to the critical revision of the manuscript. MN contributed to the management of the study. GK contributed to collection of the data. MB contributed to collection of the data. FP contributed to collection of the data. SC contributed to collection of the data. HC contributed to collection of the data. JLP contributed to the critical revision of the manuscript. YEC contributed to the conception, design, interpretation and analysis of data and drafting the manuscript.
